# Synthetic data in medical research

**DOI:** 10.1136/bmjmed-2022-000167

**Published:** 2022-09-26

**Authors:** Theodora Kokosi, Katie Harron

**Affiliations:** Population, Policy, and Practice Department, UCL Great Ormond Street Institute of Child Health, London, UK

**Keywords:** Education, medical, Information technology, Medical informatics, Statistics

**Key messages**

- Synthetic data are artificial data that can be used to support efficient medical and healthcare research, while minimising the need to access personal data
- More research is needed to determine the extent to which synthetic data can be relied on for formal analysis, the cost effectiveness of generating synthetic data, and how to accurately assess disclosure risk

Synthetic data have the potential to improve medical research while minimising the need to access personal data; Theodora Kokosi and Katie Harron explain what they are and how they are used.

## Introduction

Demand to access high quality data at the individual level for medical and healthcare research is growing. Electronic health record data collected on whole populations can help to generate real world evidence and can be used for a range of secondary purposes, including testing new hypotheses and developing and evaluating different methodological and statistical approaches. Secondary analysis of primary research data, such as from clinical trials,^1^ is also valuable—for example, to conduct meta-analyses of individual participant data. However, several complex privacy requirements make accessing these data challenging.^2^

Information contained in electronic health records or in clinical trial data are highly sensitive and access to these datasets can be an expensive and lengthy process.^3^ Data privacy and protection regulations are the main barriers to accessing these data for healthcare and medical research.^4^ Anonymisation (where potentially identifiable variables are removed) is one way to make data available; however, intensive anonymisation can degrade the data to the extent that it is no longer fit for purpose.^5^ For example, adding random noise to the data reduces precision and leads to larger confidence intervals. Several reidentification attempts on anonymised data have been successful and have harmed public and regulators’ trust in such methods.^6 7^ For instance, one study showed that patients could be identified by matching information from patient level data that was publicly available, attributing information obtained from newspapers, and contacting those patients directly.^6^

Use of information from clinical trials and electronic health records of large populations has the potential to benefit medical and healthcare research and makes seeking new approaches to data access imperative. One solution is to use so-called synthetic data, or artificial data, which provide a realistic representation of the original data source. Synthetic data look like the original data source, without containing any information on any real individuals. Synthetic data can attempt to preserve some of the statistical properties of the original data source (eg, distributions of continuous data, proportions of categorical data, correlations between variables, and other model parameters).

## Approaches to generating synthetic data

The aim of data synthesis is to create a dataset that resembles the original individual level data, and retains the same sample size, with rows for each participant and columns for each variable. Characteristics of the original data, including missing values and patterns, are replicated depending on the method chosen to generate the synthetic data. Several methods can be applied for generating such data. In medical research, machine learning methods have predominantly been used, given the complex and high dimensional nature of patients’ data. Machine learning methods for constructing synthetic data from the original data sources are typically based on generative models. These models are built to capture and accurately estimate the correct correlations and distributions between different variables in the original data source. Additionally, the models draw on inferences from the original data using bayesian networks via sampling techniques or deep learning via neural networks,^5^ such as generative adversarial networks.^8 9^ Generative adversarial networks have become particularly popular for use in synthetic data and are used to generate not only synthetic samples but also synthetic images (versions of medical images produced by a wide range of imaging methods) and image translations (conversion of one image representation to another image representation (eg, a grayscale photo to a coloured photo).^10^ These techniques attempt to generate synthetic data while dealing with privacy issues as well as patient data that are imbalanced, biased, or from a small sample.^11^ However, correction of imbalances can also worsen model performance by leading to poor calibration of risk predictions or wrong absolute risks.^12^ Alternative approaches to generative adversarial networks have also been developed more recently, such as ADS-GAN (anonymisation through data synthesis using generative adversarial networks), PATE-GAN (private aggregation of teacher ensembles), and Time-GAN (time-series generative adversarial networks).^13 14^

## Uses and benefits of synthetic data

Some of the most valuable uses for synthetic data are developing code or conducting preliminary hypothesis generation and testing before deployment in real datasets. Researchers can then develop and validate methods for a particular task before accessing real data. This process saves time because data access applications can be conducted in parallel or while waiting for data access to be granted. Synthetic data also help to preserve privacy because the amount of time that researchers need to access sensitive patient information is reduced. This type of data can also be used to improve the reproducibility of research because synthetic datasets can readily be shared with other researchers or third parties to verify models and analysis strategies.^4^ Synthetic data can also be used to accelerate methodological developments in medical research and facilitate training and capacity building in methods for handling medical data that are high dimensional and challenging to model. Additionally, synthetic data could be a solution to researchers who are already synthesising clinical study evidence. For example, researchers of a meta-analysis of individual participant data using sufficient statistics from aggregate data and who want to combine data from trials that provide individual participant data in addition to from those that do not.^15^ Similarly, synthetic data could be used in simulation studies for sample size calculations for a meta-analysis of individual participant data to account for previous knowledge (eg, number of studies promising individual participant data) in the information available.^16^

**Figure 1:**
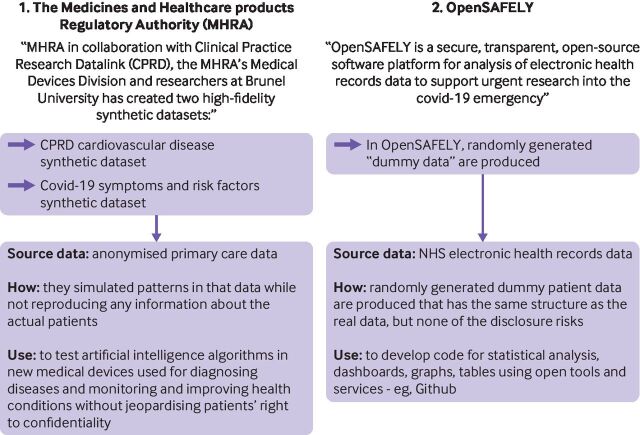
Examples of synthetic data in medical research.^20 21^

Figure 1 presents two examples of how synthetic data are being used in medical research.

## Evaluation of synthetic data

**Figure 2:**
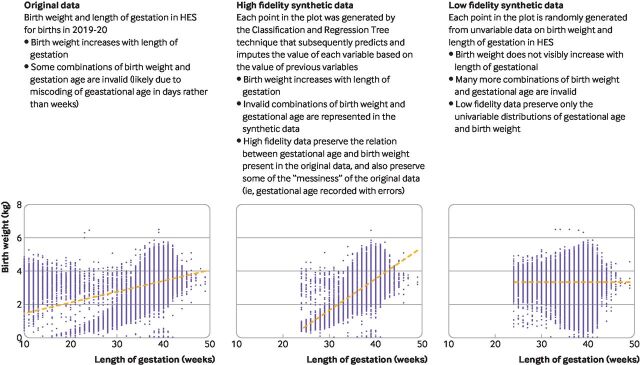
Examples of high and low fidelity synthetic data. In this example, values of birth weight and length of gestation recorded on birth records in Hospital Episode Statistics (HES) data were used to illustrate high fidelity and low fidelity synthetic data. The lines on the scatterplots represent the regression lines for birth weight on length of gestation.

Understanding how closely synthetic data replicate original data sources is vital for understanding what the data can be used for; a factor that can be thought of in terms of fidelity.^17^ Figure 2 shows the difference between low fidelity data (which do not preserve associations between different variables) and high fidelity data (which do preserve these associations). Low fidelity data can be useful for educational purposes (eg, methodological and software education) and initial data exploration, whereas high fidelity data are more useful for developing models.

The extent to which the synthetic data resemble the original data can be measured in several ways. Metrics include data usefulness, which evaluates the extent to which synthetic data resemble the statistical properties of the original data, and information disclosure, which measures how much of the real data can be shown by the synthetic data.

**Figure 3:**
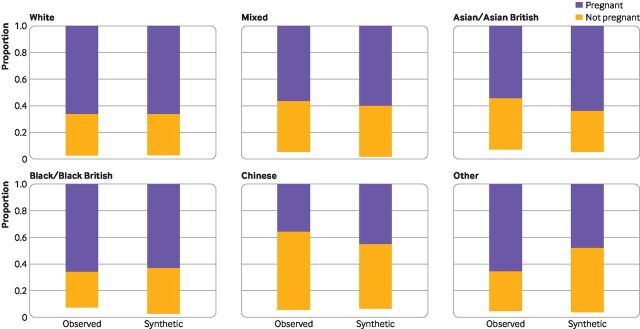
Example of visual comparison of bivariate distributions between original(observed) and synthetic data. This example is obtained from an analysis of the third National Survey of Sexual Attitudes and Lifestyles (Natsal-3)^19^ and shows the proportion of the female survey respondents who answered that they had ever been pregnant, by ethnic group. The method used to generate the synthetic data was Classification and Regression Tree (known as CART).

Approaches for measuring data usefulness include comparing univariate or multivariable distributions of variables between the original and synthetic data, or comparison of model parameters and estimates for multivariate or multivariable models, and interval overlap of confidence intervals.^18^ Figure 3 gives an example of a bivariate comparison between original (observed) and synthetic data. The similarity can also be measured between the relative performance of two algorithms (trained and tested) on the synthetic data and their relative performance (when trained and tested) on the original data.

To evaluate disclosure risk, two concepts are considered: identity disclosure, which refers to the risk of an intruder identifying an individual within a sensitive dataset, and attribute disclosure, which refers to the risk of an intruder identifying an individual based on other sensitive attributes of a patient record (eg, medical tests and diagnoses).^10^ Several methods can quantitatively assess disclosure risk and attribute disclosure, such as hamming distance, targeted correct attribution probability, and correct relative attribution probability.^10 19^

## Challenges and future directions

Although synthetic data methods were introduced more than 30 years ago, these data are not yet widely used in medical and health research, and are associated with several challenges. One area of concern is whether synthetic data would ever be used for decision making or whether final analyses will always need to be conducted on the original data.^4^ Furthermore, disclosure risk is minimised in synthetic datasets. However, the risk of including even a small number of unique observations, owing to the nature of the health data (ie, rare diseases or outliers), can pose an additional challenge to attribute disclosure. This challenge involves accurately estimating the high dimensional distribution of these data without replicating the information of the individual. Furthermore, additional research is also needed to understand the cost effectiveness of generating synthetic data—that is, whether potential benefits outweigh the time and effort required to generate synthetic data that are fit for purpose.

## Data Availability

No data are available. Authors do not have permission to share patient level Hospital Episode Statistics data. Hospital Episode Statistics data are available from the NHS Digital Data Access Advisory Group (enquiries@nhsdigital.nhs.uk) for researchers who meet the criteria for access to confidential data.
